# Increased Demand of Obese Women for Protectins, Maresin, and Resolvin D1 in the Last Trimester of Pregnancy

**DOI:** 10.3390/nu15204340

**Published:** 2023-10-11

**Authors:** Małgorzata Szczuko, Natalia Szwec-Nadworna, Joanna Palma, Małgorzata Tomasik, Maciej Ziętek

**Affiliations:** 1 Department of Human Nutrition and Metabolomics, Pomeranian Medical University, 71-460 Szczecin, Poland; natalia.szwec-nadworna@wp.pl; 2 Department of Biochemical Sciences, Pomeranian Medical University, 71-460 Szczecin, Poland; jpalma@pum.edu.pl; 3 Department of Integrated Dentistry, Pomeranian Medical University, 70-111 Szczecin, Poland; malgorzata.tomasik@pum.edu.pl; 4 Department of Perinatology, Obstetrics and Gynecology, Pomeranian Medical University, 72-010 Police, Poland; maciej.zietek@pum.edu.pl

**Keywords:** pregnancy, inflammation, EPA, DHA, resolvins, maresin, protectin

## Abstract

Background: Pregnancy is a physiological state during which inflammation occurs. This complex biological response is necessary for the implantation process as well as delivery. In turn, its suppression during gestation favors the normal course of the pregnancy. Therefore, the presence of pro-resolving mediators, EPA and DHA derivatives, The aim of this study was to investigate the changes in the levels of anti-inflammatory resolvins and their precursors in different trimesters of pregnancy with consideration of the women’s weight, including overweight and obese women before pregnancy. Methods: A total of 78 women participated in this study; the mean age and BMI before pregnancy were 32.3 ± 5.52 and 27.73 ± 6.13, respectively. The patients were divided into two groups, considering their BMI before pregnancy. The extraction of eicosanoids was performed by high-performance liquid chromatography. The results obtained were subjected to statistical analysis. The levels of all studied parameters showed statistically significant differences between the study group (SG) and the control group (CG) in the different trimesters of pregnancy. Over the course of pregnancy, the levels of protection (PDX), maresin, resolvins (RvD1, RvE1), and their precursors differed in relation to the trimester of pregnancy and the division into groups considering the correct body weight before pregnancy. Results: Overweight or obese women had significantly lower levels of RvE1 in the third trimester and their precursors compared to normal-weight women. While the levels of PDX and RvD1 were significantly higher, this may be due to both a lower intake of products rich in omega-3 fatty acids by obese women and an increased need of obese women’s bodies to quench chronic inflammatory processes associated with obesity. Conclusions: Both EPA and DHA derivatives are involved in calming down inflammation during pregnancy, which was observed.

## 1. Introduction

### 1.1. Sources of EPA and DHA

Resolvins are compounds derived from polyunsaturated fatty acids of the omega-3 (*n*-3) family. We distinguish resolvins of the D-series, which are derivatives of docosahexaenoic acid (DHA), and resolvins of the E-series, which are derivatives of eicosapentaenoic acid (EPA). Omega-3 fatty acids belong to exogenous substances, so they or their precursors must be supplied by food. The main sources of these acids are fish oils, seafood, and plant products. The oils richest in EPA and DHA are those from Atlantic menhaden, salmon, sardines, and cod liver [1]. Flaxseed oil and walnut oil can serve as vegetarian sources of EPA, whereas the extraction of DHA from plant products is more complicated. α-linolenic acid (ALA) is a precursor to the formation of DHA acids, among others. Unfortunately, studies indicate that ALA is only to a small extent converted to DHA in the human body. However, marine algae oils may be a good direct source of vegetable DHA [2]. The European Food Safety Authority (EFSA) guidelines indicate that pregnant women should increase their intake of fish because of the *n*-3 acids they contain, as these are beneficial for the proper course of pregnancy [3]. However, it should be remembered that fish (especially marine fish) may not only contain health-promoting components. Unfortunately, they are often also contaminated with mercury (especially methylmercury, MeHg), dioxins, and polychlorinated biphenyls [4]. These compounds are toxic to the human body and have carcinogenic and neurotoxic effects. Their high intake during pregnancy may affect abnormal fetal development and cause the occurrence of malformations, fetal growth restriction, and the occurrence of neurological defects [5]. Therefore, it is recommended for pregnant women to consume fish 2–3 times a week, which is a safe amount and does not threaten the health of the fetus. Supplementation with omega-3 fatty acids is also worth considering if a pregnant woman is not able to cover her requirements with a diet from a safe source only [6]. The recommended amount of fatty acids supplied is 250 mg/day of DHA + EPA [7]. 

### 1.2. Inflammation in Pregnancy

Pregnancy is a physiological state in which inflammation develops to condition vascular remodeling, embryo implantation, and delivery. A significant increase in leukocytes, prostaglandins, and cytokines has been noted during the implantation process [8]. This is because the genetic material of the embryo contains not only the mother’s genetic information but also that of the father. It is the latter that provides the signal to the body to start the body’s defense response and ultimately generate inflammation. [9]. Initiating an inflammatory response allows the placenta to develop, allowing vessel remodeling [10]. An important difference between classical inflammation and implantation-associated inflammation is that neutrophils are not involved in implantation, preventing a full immune response [11]. Proinflammatory cytokines produced by the embryo induce an inflammatory response in the endometrium, which increases the expression of genes necessary for embryo adhesion during the implantation window [12]. The second important moment during which inflammation plays a crucial role during normal pregnancy is the last period of pregnancy and the postnatal period. The inflammatory response during labor may be helpful for removing fragments of the placenta and making the uterus ready for the pathogens that are present in the immediate postpartum period. Under normal conditions, the inflammatory reaction weakens during the first two trimesters of pregnancy, and it develops from the beginning of the last trimester [13,14].

### 1.3. Synthesis of Resolvin

The *n*-3 fatty acid derivatives are otherwise known as specialized pro-resolving mediators [14]. This group includes resolvins (derivatives of EPA and DHA acids), neuroprotectins (DHA derivatives), and maresins (also DHA derivatives) [15]. They are present in the fluids, tissues, and cells of the mother’s body. Acetylated cyclooxygenase-2 (ASA-COX2) participates in the synthesis of resolvins. This enzyme is formed from acetylsalicylic acid (ASA) and cyclooxygenase-2 (COX-2) [16]. Cyclooxygenase (COX) is an enzyme that exists in two isoforms: cyclooxygenase-1 (COX-1) and cyclooxygenase-2 (COX-2). Among other activities, COX-2 is responsible for the development of the inflammatory response throughout the synthesis of pro-inflammatory prostaglandins [17]. 

Another enzyme important in the synthesis of resolvins is 5-lipooxygenase (5-LOX). It is one of the isoforms of lipoxygenase (LOX). It belongs to the family of non-heme iron-containing dioxygenases [18]. Its activity is regulated by the 5-Lipoxygenase-activating protein (FLAP), which acts by protein reaction. The complex of FLAP and 5-LOX produces 5-HEPE (5-hydroEPE) [19].

### 1.4. E-Series Resolvins

**Figure 1:**
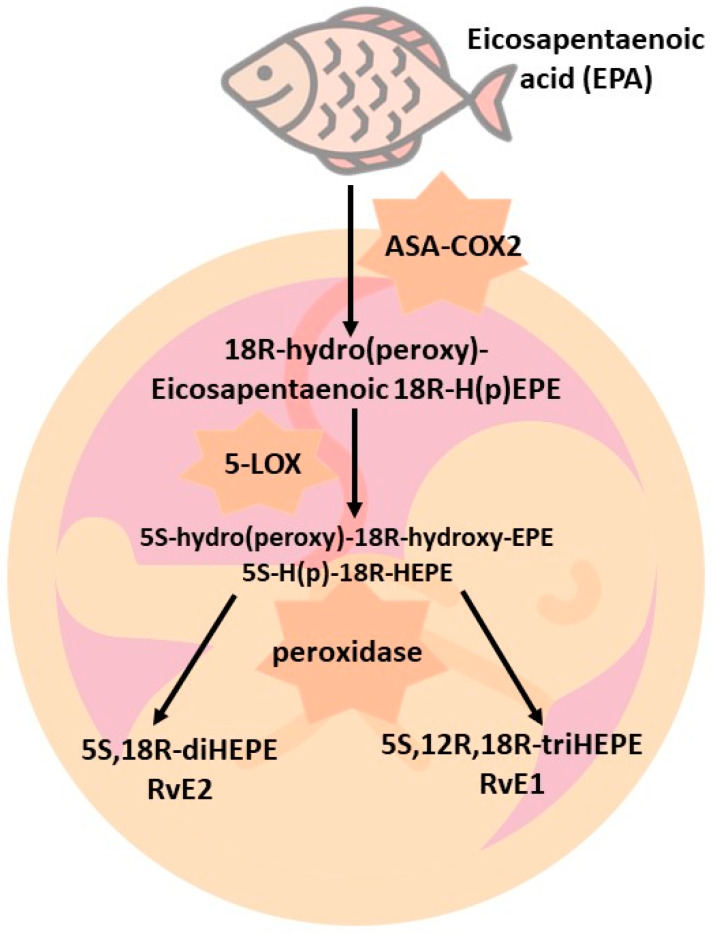
Synthesis of E-series resolvins.

EPA acid is catalyzed by the enzyme ASA-COX2 to form 18R-H(p)EPE (18-hydro(peroxy)-eicosapentaenoic acid). In the presence of 5-LOX, 18R-H(p)EPE is converted to 5S-H(p)-18R-HEPE, then to 5S(6)-epoxy-18R-HEPE, and finally to 5S,12R,18R-triHEPE, called resolvin E1 (RvE1) [20]. On the other hand, if 18R-H(p)EPE is transformed to 5S-H(p)-18R-HEPE, then 5S, 18R-diHEPE, or resolvin E2 (RvE2), is obtained [21,22]. Several resolvins exhibit specific regulatory functions. For example, RvE1 regulates leukocyte adhesion molecules [22,23], ADP-dependent platelet activation [24], and PMN (polymorphonuclear leukocyte, or granulocyte; PMN) apoptosis to accelerate catalase and resolve inflammation [25,26]. A scheme for the synthesis of E-series resolvins is shown in Figure 1. 

The main receptor for E-series resolvins is the ChemR23 receptor (the G protein-coupled receptor for RvE1) [20]. ChemR23 belongs to the GPCR (G protein-coupled receptor) group. It can also be found under the name CMKLR1 (chemokine-like receptor 1) [27]. Chemokines, otherwise known as chemotactic cytokines, are proteins that “attract” leukocytes by chemotaxis (the closer to the focus of inflammation, the greater the gradient of chemokine concentration) [28]. After attachment to ChemR23, RvE1 promotes the resolution of inflammation by enhancing phagocytosis of apoptotic neutrophils by macrophages or inhibiting T-lymphocyte chemotaxis [29]. It also inhibits the production of proinflammatory cytokines by blocking TNF-α-induced NF-kB (nuclear factor kappa-light-chain enhancer of activated B cells) signaling, which is responsible for cytokine production [30]. Thus, it can be concluded on this basis that RvE1 acts antagonistically to chemerin. Interestingly, in a study by Spanish investigators, the rs1878022 SNP (single nucleotide polymorphism) was discovered in the gene encoding ChemR23. Individuals carrying the T allele tended to be obese, whereas those carrying the C allele were normal-weight individuals who additionally showed increased ChemR23 expression in visceral adipose tissue, significantly lower levels of inflammatory cytokines and chemokines, and reduced inflammation in adipose tissue compared to individuals carrying the other allele of the gene [31]. Resolvin E2 is not a ligand for the ChemR23 receptor. In contrast, the receptor for RvE2 has not yet been identified, and further studies in this direction are needed [32]. 

### 1.5. D-Series Resolvents

**Figure 2:**
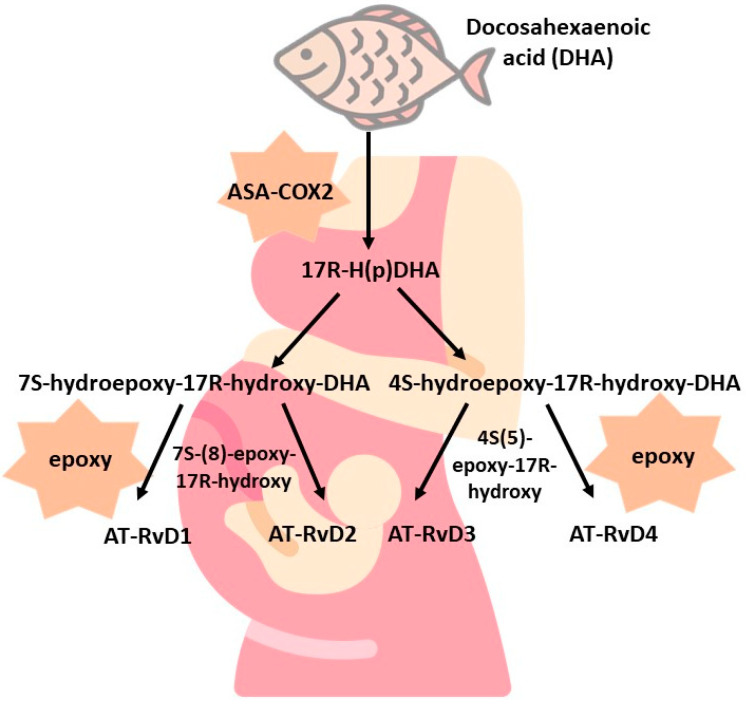
ASA-COX-dependent synthesis of D-series resolvins.

**Figure 3:**
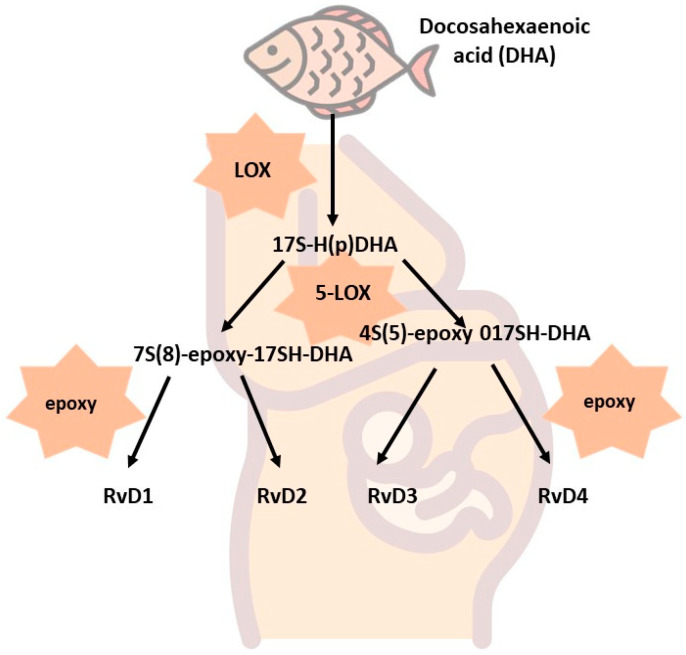
LOX-dependent synthesis of D-series resolvins.

D-series resolvins can be formed by two routes, differing slightly in the reactions that occur. The first is ASA-COX-dependent and ends with AT-Rv-D1—AT-RvD4 resolvin synthesis. DHA is converted with ASA-COX to 17R-H(p)DHA and then with 7s-H(p)-HDHA, and 7S(8)-epoxy-HDHA transforms into AT-RvD1 and AT-RvD2 resolvins. On the other hand, if the reaction occurs with 4S-H(p)-HDHA and 4S(5)-epoxy-HDHA, the products of the reaction will be AT-RvD3 and AT-RvD4 resolvins. A LOX-dependent reaction can also appear. Then, the formed 17S-H(p)DHA in the presence of 5-LOX is converted into 7S(8)-epoxy-17SH-DHA to give resolvins D1 and D2 (RvD1 and RvD2) and into 4S(5)-epoxy-17SH-DHA, which is further converted into resolvins D3 and D4 (RvD3 and RvD4) [20]. The schemes for the synthesis of E-series resolvins are shown in Figure 2 and Figure 3. 

D-series resolvins also have specific receptors. ALX/FPR2 (lipoxin A4/formyl peptide receptor 2) is an example of a receptor for RvD1 and RvD3 [32]. The name ALX comes from the fact that the main ligand for this receptor is lipoxin 4A (LXA4) [33]. Another receptor for RvD1 and RvD3 is DRV1/GPR23 (G protein-coupled receptor). It can also be a receptor for resolvins synthesized with ASA-COX2 or AT-RvD resolvins [34]. RvD1 binds to both receptors antagonistically, depending on the needs of the organism. It acts on DRV1 when the body maintains homeostatic processes, while it acts on ALX/FPR2 in response to an inflammatory stimulus to quench inflammation [35]. At the same time, DRV2/GPR18 is a receptor for RvD2. 

Based on the available studies, resolvins are found to play an important role during pregnancy, labor, and the postpartum period. In 2013, Jones et al. tested the effects of feeding rats a diet rich in high omega-3 fatty acids in the presence of SPM in the maternal placenta. The ingestion of the fortified diets by the rats in the control group clearly showed a significant increase in 18-HEPE and 17-HDHA levels. The levels of RvD1 and RvD2 resolvins also increased, but only at the end of pregnancy. On the other hand, the levels of IL-6 and TNF-α, which are pro-inflammatory factors, were significantly lower than in the control group [35]. 

The presence of resolvins and their precursors has also been studied in the human placenta. In 2015, a study analyzing the effect of omega-3 fatty acid supplementation on changing the levels of PUFAs, resolvin D-series, and their precursors in the placenta of pregnant women was published. The results showed that after *n*-3 supplementation, the levels of DHA (by about 80%) and the precursors for resolvins 17-HDHA and 18-HEPE increased significantly in the placenta, which indicates the participation of these mediators in the human placenta. In contrast, the levels of EPA, RvD1, and RvD2 were similar to those in CG [36]. A recent study by Ulu A. et al. in 2019 investigated the female placenta as a potential target for SPM action, reporting a significant increase in SPM in maternal blood compared to cord blood. To test the validity of their hypothesis, the investigators performed immunohistochemistry for the RvD2 receptor GRP-18 in the placenta. It turned out that the expression of this receptor was very high in the smooth muscles of placental blood vessels and the extravascular trophoblasts of placental tissues. This allows us to speculate that the placenta is indeed a target of specialized anti-inflammatory mediators [37]. 

Supplementation with omega-3 fatty acids may not only have a positive effect on pregnancy but also exert beneficial changes in the development of newborns. See et al. conducted a study in which they examined how omega-3 fatty acid supplementation during pregnancy and after delivery would increase anti-inflammatory resolvins in the children of these mothers [38]. In this study, the results confirmed a three-fold increase in 18-HEPE levels and a 1.5-fold increase in 17-HDHA levels compared to the control group. 18-HEPE and 17-HDHA are precursors of D-series and E-series resolvins in cord blood. However, for the same children at 12 years of age, the results did not differ between groups [38]. Another study examining the effect of fatty acid supplementation on infants from birth to six months of age yielded similar results. Compared to the control group, infants taking supplementation had significantly higher levels of 18-HEPE; however, when supplementation was stopped and six months had passed since then, no significant differences were seen between the two groups [39]. This leads to the conclusion that resolvin levels may be modified by supplementation; however, the long-term effect of this correlation is not sustained. The level of the precursor for D-series resolvins, 17-HDHA, in cord blood is significantly higher during natural vaginal childbirth, which requires activation of inflammatory pathways than compared to cesarean delivery [40]. Elevated levels of RvD1 and RvE1 were also determined in the breast milk in the Swiss study. The results of the investigation indicated that the levels of 18-HEPE, a precursor for RvE1, during the first month of lactation, are at similar levels, and they fall within the concentration range of 3–6 ng/mL. On the other hand, levels of 17-HDHA, the precursor for RvD1, drop from about 40 ng/mL in the first week to about 20 ng/mL in week four of lactation. Resolvin levels themselves also change over the first month of lactation. However, the authors indicate that both resolvins and their precursors are significantly higher in breast milk than in plasma. Therefore, it can be concluded that, owing to resolvin content, among others, breast milk plays a key role in building the immunity of the newborn and is the reason for the advantages of using breast milk over formula milk [41]. Studies also indicate that *n*-3 supplementation by the mother may affect the absence of allergy and atopy symptoms in her infant [42]. The authors of this study decided to check whether obesity in pregnant women affects the level of SPM of EPA and DHA long-chain fatty acids. And whether it worsens the process of extinguishing inflammation in the course of pregnancy. 

## 2. Materials and Methods

### 2.1. Characteristics of the Study Group

A total of 78 Caucasian pregnant women, aged 32.3 ± 5.52, were included in the investigation. The study group (SG) consisted of overweight and obese women with a BMI > 25 before pregnancy. The control group (CG) consisted of women with a normal pre-pregnancy weight and a BMI < 25. All pregnancies were singleton and delivered at or near term (≥38 weeks gestation). Exclusion criteria included disorders that would preclude informed consent to participate in this study, the presence of active infection or malignancy, or complicated pregnancies. The characteristics of this study group and control group are shown in Table 1. 

The two groups did not differ in age and height. The women in this study group had significantly higher insulin and fasting glucose concentrations. They also had higher values of triglycerides and LDL-fraction cholesterol compared to the control group. In contrast, patients in the control group had significantly higher HDL cholesterol and higher total cholesterol concentrations. 

Patients from each group were divided into three subgroups depending on the stage of pregnancy: the first, second, and third trimesters. The first trimester takes place from conception through week 13. The second trimester is from week 14 through week 28. The third trimester is from week 28 through labor and delivery. In this study group, the number of women in the subgroups was as follows: first trimester, 21 women; second trimester, 20 women; third trimester, 7 women. However, in the control group, the number of women in each trimester was 10. 

All patients were educated on proper nutrition by qualified clinical dietitians. All patients received 14 sample menus, taking into account the needs of pregnant women based on the Nutrition Standards for the Polish population [43]. 

### 2.2. Research Methods and Tools

After adequate stem cell preservation, 5 mL of blood was collected from each patient. The samples were placed in tubes containing arsenic acid and centrifuged using a refrigerated centrifuge. After centrifugation was complete, the separated plasma was collected into Eppendorf Tubes^®^ 5.0 mL. These tubes were stored at −80 °C until biochemical analysis.

#### 2.2.1. Chemical Reagents

The reagents used in this study were purchased from Merc KGaA (Warsaw, Poland). All were of a purity grade suitable for high-performance liquid chromatography (HPLC). The buffers that were used for HPLC analysis were filtered through 0.22 µm nylon filters (Agilent). Double-distilled water was obtained from the Milli-Q Water System (Millipore, Billerica, MA, USA). 

#### 2.2.2. Eicosanoid Extraction and HPLC Operating Parameters

Rev D1, Rev E1, Maresine1, 18-HEPE, and 10(S)17(R)DiDHA (Protectin DX) were extracted from plasma using an RP-18 SPE column extraction column (Agilent Technologies, Cheadle, UK). The method described in detail in [44] was used. HPLC separation was performed using an Agilent Technologies 1260 liquid chromatograph. Agilent ChemStation software was used for instrument control, data acquisition, and analysis. Quantitative analysis was performed using ChemStation Software (Agilent Technologies, Cheadle, UK) [45]. 

### 2.3. Statistical Analysis

Statistical analysis was performed using the Statistica 13.3 program (Statsoft, Krakow, Poland). If the distribution was normal, parametric tests (the Shapiro–Wilk test) were applied. Otherwise, non-parametric tests (the Mann–Whitney test) were used. The significance level was defined as *p* < 0.05.

## 3. Results

Plasma CG was determined for RvE1, RvD1, Maresin 1, and the precursors 10S17R DiHDHA, 18RS HEPE, and 17RS HDHA. The significance of the difference in levels in each trimester in both connected groups is shown in Table 2. The results obtained for CG are shown in Table 3, while those for SG are shown in Table 4. Table 5 shows a comparison of individual trimesters between CG and SG. 

### 3.1. Comparison of Trimesters in the Group of All Patients

Comparing the values of both groups together, significant differences were found in the parameter levels between the second and third trimesters (RvE1, 18RS HEPE), and the trend was also seen in the levels of other metabolites (RvD1, PDX, Maresin 1). The differences between the first and third trimesters were even more pronounced. A significant decrease in the level was noted for RvD1, Maresin 1, and 18RS HEPE, while the trend was noticeable for RvE1 and 17RS HDHA (Table 2). Significant differences between the first and second trimesters were not observed. 

### 3.2. Comparison of Trimesters within a CG

In the control group, both resolvin RvE1 and RvD1 levels increased between the first and third trimesters and the second and third trimesters (Table 3). Significant differences between the first and second trimesters in the control group were not observed. Maresin 1 decreased in blood level in each trimester. The levels of PDX and 17RS HDHA increased between the second and third trimesters; the trend was visible, but no significant differences were found (Table 3). 

### 3.3. Comparison of Trimesters within a SG

In this study group, one significant relationship was found that concerned the increase in the level of 18RS HEPE both in the second and third trimesters. There was a noticeable decrease in the level of both resolvins; however, the changes were not statistically significant (Table 4). 

### 3.4. Comparison of Trimesters between Groups

The comparison of trimesters between groups SG and CG showed a significant difference in the first trimester only with respect to 18RS HEPE. The level of 18RS HEPE was lower in the SG group (Table 5). In the second trimester, two significant differences were found in relation to 18RS HEPE and 17RS HDHA, the levels of which were lower in the SG group. In the third trimester, no significant statistical differences were observed between the SG and CG groups. The trend was noticeable for both resolutions (Table 5). 

### 3.5. Weight Increase during Pregnancy

The correlation (*p* < 0.05) of body weight gain in individual study groups (All, SG, and CG) was checked. It was found that excess body weight during pregnancy had no significant effect on the level of the studied mediators (Table 6). 

## 4. Discussion

This study investigated the parameters of pregnant women at different stages of pregnancy, taking into account the presence of overweight and obesity before pregnancy. A comparison of these parameters with those of the control group allows us to draw conclusions about the involvement of EPA and DHA derivatives. The sparse literature on this topic has made this study pioneering in the field, especially if we factor in pre-pregnancy obesity. 

In the study group (SG), which was characterized by increased body weight before pregnancy, the levels of RvD1 and their precursors were significantly lower than in the group of normal-weight women, which may confirm previous observations that obesity increases the chronic inflammatory process [46] and thus increases risk during pregnancy. This probably results from the fact that obesity is associated with the endocrine activity of adipose tissue [46,47]. The secretion of inflammatory cytokines such as tumor necrosis factor-α (TNF-α) and interleukin 6 (IL-6) by adipocytes is increased in obesity-induced adipose tissue inflammation. 

On the other hand, an Australian-Iranian study compared RvD1 levels in women with polycystic ovary syndrome (PCOS), which, like obesity, is characterized by increased inflammation. The results of the cited studies indicate higher levels of RvD1 in women with PCOS compared to healthy women [48]. The reason for this result may be that these women consumed significantly more omega-3 fatty acids, from which RvD1 is synthesized. The researchers concluded that properly planned and tailored supplementation with omega-3 fatty acids may improve the synthesis of resolvins in the presence of PCOS, which appears to also improve the situation for obese pregnant women. In our studies, no significant differences in resolvin levels were observed; however, most pregnant women supplemented with omega 3, which could prevent the reduction of resolvin levels. In contrast to the RevD1 described, the level of Rev E1 in both groups (SG and CG) decreased in the third trimester. Moreover, its precursor 18RS HEPE was systematically increasing; therefore, it can be concluded that the synthesis was intensified in both groups, but probably too low in relation to the demand. A similar situation is in the case of the precursor 17RS HDHA, whose presence increases in the CG group but remains at a similar level in the SG. It is therefore likely that in the obese SG group, the enhanced synthesis of the Rev D1 pathway took place earlier; therefore, we do not observe it during pregnancy. An Australian study [49] examined the frequency of intake of specific products among normal-weight and increased-weight women who did not have any comorbidities. This study used the Food Frequency Questionnaire (FFQ) and examined blood fatty acid levels. This study indicated that despite similar dietary intake of omega-3 fatty acids, blood levels of these fatty acids were significantly lower in obese women than in healthy women. Therefore, we are inclined to conclude that obese individuals have an increased need for omega-3 fatty acids as a result of ongoing low-grade chronic inflammation, which was observed as a trend in the third trimester relative to RvE1. And that is why changes in synthesis during pregnancy are different than in women with a normal body weight before pregnancy. 

As communicated by other researchers, RvD1 resolvin levels increase when pregnancy is threatened [50]. The results obtained in the present study indicate that women in the control group (CG) had significantly higher levels of RvD1 compared to this study group, which in our opinion may be explained by the mediator’s activity in the anti-inflammatory process in the group of obese women. This result is significant because the elevated body weight that this study group had may be a predisposing factor for the development of gestational pathology. My own research showed a slightly different relationship, which probably resulted from faster use of the mediator in the phase of earlier inflammation and thus the exhaustion of the body’s buffering mechanisms. Results similar to ours were obtained in another study where the level of RvD1 in women with preterm and normal labor was examined. The reported findings showed that RvD1 levels were significantly higher in women with term deliveries compared to mothers of preterm infants. The results obtained in the above study indicate a similar trend in that excessive body weight characterizing SG is one of the predisposing factors for preterm delivery. 

It is also worth noting the differences in levels of PDX; an increase in levels in the third trimester was observed only in women with a normal body weight before pregnancy (CG). The most difficult to interpret are the results concerning maresins. It seems that their participation is indisputable; however, due to the relatively small number of patients in the subgroups, the relationships were difficult to observe. Unfortunately, there are no studies examining the effect of this resolvin level on pregnancy in either obese or normal-weight women. To date, levels of resolvin precursors throughout pregnancy have not been examined in any other study. In the current study, the levels of these precursors were significantly higher in SG, which could be due to the increased synthesis of inflammation-quenching mediators that were observed in obese individuals [51]. 

## 5. Conclusions

The results of this study support the hypothesis that EPA and DHA derivatives (protectins, maresin, and resolvin D1) and their precursors are involved in suppressing the inflammatory reaction during pregnancy, especially in the last trimester. Moreover, the level of DHA derivatives is lower in obese women and overweight women in this period with a higher level of precursors, while EPA derivatives are significantly lower in the group of women who had a higher body weight before pregnancy compared to normal-weight women. This may be caused both by a lower intake of products rich in omega-3 fatty acids by obese women and by an increased demand from their bodies. At this point, it is difficult to determine whether weight gain during pregnancy is more important than high pre-pregnancy weight or vice versa, which is a limitation of this study. Despite the limitations of this study due to the study group size in each trimester divided into CG and SG groups, significant differences were shown. The significance between the two groups indicates that research in this field should be continued and developed so that future findings can be used in the care of pregnant patients.

## Figures and Tables

**Table 1:** Characteristics of both groups (SG and CG).

Parameter	SG Avg ± SD	CG Avg ± SD	*p*-Value
Age (years)	31.92 ± 5.38	32.6 ± 5.79	0.9257
Height (m)	1.68 ± 0.05	1.67 ± 0.05	0.3335
Body weight (kg)	94.51 ± 15.68	60.87 ± 6.61	<0.0001
BMI (kg/m^2^)	32.01 ± 9.11	21.73 ± 2.24	<0.0001
Weight gain (kg)	7.97 ± 3.94	14.85 ± 4.75	<0.0001
Glucose (mg/dL)	81.92 ± 9.58	74. 91 ± 14.7	<0.0001
Insulin (mU/mL)	18.51 ± 14.2	14.35 ± 22.9	0.0002
Total cholesterol (mg/dL)	198.41 ± 53.9	203.01 ± 48.7	0.4866
Cholesterol HDL (mg/dL)	67.03 ± 14.7	76.21 ± 11.7	0.0077
Cholesterol LDL (mg/dL)	125.71 ± 39.6	123.77 ± 43.1	0.6926
Triglycerides (mg/dL)	161.39 ± 71.8	138.22 ± 54.5	0.4340
Number of women	48	30	-

SG: study group; CG: control group; SD: standard deviation.

**Table 2:** Comparison of mediator levels in the group of all patients.

Parameter	I Trimester Avg ± SD	II Trimester Avg ± SD	III Trimester Avg ± SD	*p*-Value I–II	*p*-Value I–III	*p*-Value II–III
RvE1	0.049 ± 0.036	0.048 ± 0.037	0.024 ± 0.027	0.954	0.053 *	0.050
RvD1	0.002 ± 0.001	0.002 ± 0.001	0.005 ± 0.011	0.434	0.046	0.056 *
10S17R DiHDHA (PDX—protection)	0.015 ± 0.010	0.012 ± 0.009	0.023 ± 0.035	0.244	0.237	0.057 *
Maresin 1	0.001 ± 0.001	0.002 ± 0.001	0.008 ± 0.020	0.707	0.045	0.051 *
18RS HEPE	0.005 ± 0.003	0.005 ± 0.004	0.021 ± 0.043	0.443	0.027	0.030
17RS HDHA	0.013 ± 0.004	0.022 ± 0.028	0.067 ± 0.171	0.116	0.058 *	0.161

RvE1: resolvin E-series; RvD1: resolvin D-series; 18RS-HEPE—18R-hydroxyeicosapentaenoic acid; 17RS-HDHA—17-hydroxy docosahexaenoic acid; CG: control group, SD: standard deviation, *—trend; resolvins; RvD—D-series resolvins; PDX—protectin DX; PD—D-protectins; Mar—maresins.

**Table 3:** Selected anti-inflammatory mediators in CG by trimester of pregnancy.

Parameter	I Trimester Avg ± SD	II Trimester Avg ± SD	III Trimester Avg ± SD	*p*-Value I–II	*p*-Value I–III	*p*-Value II–III
RvE1	0.047 ± 0.036	0.049 ± 0.048	0.019 ± 0.028	0.934	0.029	0.033
RvD1	0.002 ± 0.002	0.002 ± 0.002	0.009 ± 0.014	0.757	0.030	0.035
10S17RDiHDHA (PDX-protection)	0.018 ± 0.014	0.011 ± 0.010	0.034 ± 0.049	0.115	0.181	0.054 *
Maresin 1	0.002 ± 0.001	0.002 ± 0.002	0.013 ± 0.028	0.791	0.078 *	0.084 *
18RS HEPE	0.008 ± 0.006	0.008 ± 0.006	0.030 ± 0.059	0.882	0.102	0.098
17RS HDHA	0.015 ± 0.008	0.038 ± 0.046	0.119 ± 0.239	0.125	0.058 *	0.143

RvE1: resolvin E-series; RvD1: resolvin D-series; 18RS-HEPE—18R-hydroxyeicosapentaenoic acid; 17RS-HDHA—17-hydroxy docosahexaenoic acid; CG: control group, SD: standard deviation, *—trend.

**Table 4:** Selected anti-inflammatory mediators in SG by trimester of pregnancy.

Parameter	I Trimester Avg ± SD	II Trimester Avg ± SD	III Trimester Avg ± SD	*p*-Value I–II	*p*-Value I–III	*p*-Value II–III
RvE1	0.049 ± 0.037	0.048 ± 0.032	0.030 ± 0.016	0.909	0.099	0.081 *
RvD1	0.002 ± 0.001	0.002 ± 0.001	0.001 ± 0.001	0.478	0.185	0.078 *
10S17R DiHDHA (PDX—protection)	0.014 ± 0.009	0.014 ± 0.009	0.013 ± 0.009	0.489	0.674	0.854
Maresin 1	0.001 ± 0.001	0.001 ± 0.001	0.003 ± 0.004	0.899	0.078 *	0.087 *
18RS HEPE	0.002 ± 0.002	0.004 ± 0.002	0.012 ± 0.017	0.487	0.028	0.040
17RS HDHA	0.013 ± 0.003	0.015 ± 0.006	0.013 ± 0.023	0.271	0.971	0.374

RvE1: resolvin E-series; RvD1: resolvin D-series; 18RS-HEPE—18R-hydroxyeicosapentaenoic acid; 17RS-HDHA—17-hydroxy docosahexaenoic acid; SG: study group, SD: standard deviation, *—trend.

**Table 5:** Comparison of mediator levels between both groups (SG and CG) by trimester of pregnancy.

Parameter	I Trimester SG vs. CG *p*-Value	II Trimester SG vs. CG *p*-Value	III Trimester SG vs. CG *p*-Value
RvE1	0.916	0.927	0.060 *
RvD1	0.623	0.699	0.069 *
10S17R DiHDHA (PDX—protection)	0.315	0.809	0.157
Maresin 1	0.511	0.433	0.232
18RS HEPE	0.002	0.008	0.322
17RS HDHA	0.230	0.026	0.137

RvE1: resolvin E-series; RvD1: resolvin D-series; 18RS-HEPE—18R-hydroxyeicosapentaenoic acid; 17RS-HDHA—17-hydroxy docosahexaenoic acid; CG: control group; SG: study group *—trend.

**Table 6:** Correlation of weight gain in the total group and between groups (SG and CG).

Parameter	All Patients	CG	SG
RvE1	0.151	0.216	−0.180
RvD1	0.112	0.178	0.222
10S17R DiHDHA(PDX—protection)	0.103	0.184	0.212
Maresin 1	0.092	0.167	0.068
18RS HEPE	0.131	0.139	0.194
17RS HDHA	0.100	0.148	0.215

RvE1: resolvin E-series; RvD1: resolvin D-series; 18RS-HEPE—18R-hydroxyeicosapentaenoic acid; 17RS-HDHA—17-hydroxy docosahexaenoic acid; CG: control group; SG: study group.

## Data Availability

The data are available on request.
